# The Hypoxia-Inducible Transcription Factor ZNF395 Is Controlled by IĸB Kinase-Signaling and Activates Genes Involved in the Innate Immune Response and Cancer

**DOI:** 10.1371/journal.pone.0074911

**Published:** 2013-09-23

**Authors:** Darko Jordanovski, Christine Herwartz, Anna Pawlowski, Stefanie Taute, Peter Frommolt, Gertrud Steger

**Affiliations:** 1 Institute of Virology, University of Cologne, Cologne, Germany; 2 Bioinformatics Core Facility, CECAD Cologne, Cologne, Germany; 3 Cologne Center for Genomics, University of Cologne, Cologne, Germany; McMaster University, Canada

## Abstract

Activation of the hypoxia inducible transcription factor HIF and the NF-ĸB pathway promotes inflammation-mediated tumor progression. The cellular transcription factor ZNF395 has repeatedly been found overexpressed in various human cancers, particularly in response to hypoxia, implying a functional relevance. To understand the biological activity of ZNF395, we identified target genes of ZNF395 through a genome-wide expression screen. Induced ZNF395 expression led to the upregulation of genes known to play a role in cancer as well as a subset of interferon (IFN)-stimulated genes (ISG) involved in antiviral responses such as IFIT1/ISG56, IFI44 and IFI16. In cells that lack ZNF395, the IFN-α-mediated stimulation of these factors was impaired, demonstrating that ZNF395 is required for the full induction of these antiviral genes. Transient transfections revealed that ZNF395-mediated activation of the IFIT1/ISG56 promoter depends on the two IFN-stimulated response elements within the promoter and on the DNA-binding domain of ZNF395, a so-called C-clamp. We also show that IĸBα kinase (IKK)-signaling is necessary to allow ZNF395 to activate transcription and simultaneously enhances its proteolytic degradation. Thus, ZNF395 becomes activated at the level of protein modification by IKK. Moreover, we confirm that the expression of ZNF395 is induced by hypoxia. Our results characterize ZNF395 as a novel factor that contributes to the maximal stimulation of a subset of ISGs. This transcriptional activity depends on IKK signaling further supporting a role of ZNF395 in the innate immune response. Given these results it is possible that under hypoxic conditions, elevated levels of ZNF395 may support inflammation and cancer progression by activating the target genes involved in the innate immune response and cancer.

## Introduction

Gene expression arrays repeatedly found the expression of the cellular factor ZNF395 (previously called PBF for Papillomavirus binding factor) significantly increased in various cancers such as renal cell carcinomas, osteosarcomas and Ewing sarcomas [1,2,3,4]. Notably, in glioblastomas and neuroblastomas, ZNF395 was among few upregulated genes that were characteristic for a hypoxic response and associated with disease outcome [5,6]. ZNF395 also belonged to the genes upregulated in various cancer cell lines by hypoxia and by overexpression of the hypoxia-inducible transcription factor-1α (HIF-1α) [7,8,9]. These data imply that ZNF395 has a functional role within pathways involved in hypoxia and cancer. However, almost nothing is known about the biological activity of ZNF395 within the cell.

Hypoxia reflecting oxygen deficiency often occurs in developing tumors and is also a characteristic feature of acute foci of tissue inflammation. Prolyl hydroxylases (PHDs) hydroxylate HIF-α subunits and target them to ubiquitin-dependent degradation. Under conditions of hypoxia, PHDs are inhibited. HIF-α subunits then accumulate and translocate to the nucleus where they dimerize with their stable partner HIF-1β, and bind to their target sequence known as hypoxia response element (HRE). HIF induces the expression of more than 100 genes regulating glucose metabolism, cell proliferation and cell survival as well as genes that drive angiogenesis and induce immune tolerance (reviewed in [10]). Aberrant activation of NF-ĸB, the key immune response regulator, is another characteristic of many cancers. It is preceded by IĸB kinase (IKK)-mediated phosphorylation of the inhibitors of NF-ĸB, the IĸBα proteins, resulting in their proteasome-dependent degradation (reviewed in [11,12]). The IKK complex is composed of the two catalytic subunits IKKβ and IKKα and the regulatory IKKγ. The NF-ĸB pathway is crucial for the innate immune response. This is initiated upon recognition of non-self pathogen-associated molecular patterns (PAMPs) which are detected by host pattern recognition receptors (PRR) such as the toll-like receptor 3 (TLR3). Upon ligand binding, i.e. viral RNA, TLR3 triggers the activation of the kinases TBK1 and IKK, leading to the phosphorylation and activation of the transcription factors IRF3 and NF-ĸB, respectively. IRF3 and NF-ĸB induce an antiviral response by increasing the expression of the type I interferons (IFN) IFN-α and IFN-β, which were secreted by the infected cells. Their binding to the type I IFN receptor stimulates the JAK-STAT pathway to activate the transcription factor ISGF3. The complex then translocates into the nucleus, binds to the IFN-stimulated response element (ISRE) and promotes the transcription of the IFN-stimulated genes (ISGs), which encode proteins with antiviral activities (reviewed in [13]). The innate immune response and the hypoxic response are connected since hypoxia not only stabilizes HIF1-α, but also stimulates the activation of NF-ĸB [14]. Activated NF-ĸB was shown to up-regulate the expression of HIF-1α [15,16,17,18]. IKKβ also reveals NF-ĸB independent pro-tumorigenic functions, comprising IKKβ-mediated phosphorylation of the tumor suppressors FOXO3a and p53, respectively, which triggers their proteolytic degradation [19,20].

We have isolated the open reading frame (ORF) for ZNF395 through its ability to bind to regulatory sequences within the control region of several papillomavirus types (PV) [21]. It was also found that the protein binds to the control region of the Huntington disease (HD) gene and was named HDBP2 (HD binding protein 2) in this study [22]. The ORF for ZNF395 is phylogenetically conserved in vertebrates. It is very similar to the GLUT4 enhancer factor (GEF, HDBP1), which activates the gene expression of GLUT4, a glucose transporter, and the mouse glucocorticoid-induced gene 1 (GIG1, the human gene ZNF704). Particularly, these proteins share the three highly conserved regions CR1, CR2 and CR3 and have the potential to form one zinc finger structure. ZNF395 was characterized as a nucleo-cytoplasmic shuttling protein [22,23]. The overexpression of ZNF395 induced cell death [24] and repressed both the HD-promoter and the human PV type 8 (HPV8) promoter, which depended on DNA binding of ZNF395 and on targeting the SIN3/HDAC1/2 complex [25,26]. We were interested in identifying other target genes of ZNF395 and understanding the control of its transcriptional activity.

## Materials and Methods

### Cell culture

The human skin cancer cell line RTS3b is described in [27] and was kindly provided by I. Leigh, London, UK. RTS3b cells were maintained in E-medium, the monocytic cell line U937 [28] in RPMI1640, U87-MG and C33A cells in DMEM, all media were supplemented with 10% FCS and antibiotics. U937, U87-MG and C33A cells were purchased from the ATCC. Transient transfections were performed with the X-tremeGENE reagent (Roche Diagnostics) according to the manufacturer’s protocol. 48 hours later luciferase activity was determined, as described previously [29]. The relative light units were normalized by the protein concentrations of the samples. Where indicated, polyI:C (Invivogen) was added at 10ng/ml for 24h, TNFα (Cell signaling) at 1ng/ml for 24h, BMS-345541 (Sigma-Aldrich) at 5µM for 24h, MG132 (Biomol) at 25µM for 24h, and IFN-α (Biomol) at 1000U/ml for 6h. The RTS3b-TR-ZNF395 cell line was generated by stably transfecting pcDNA6/TR, followed by a pcDNA4/TO vector (both from Invitrogen) encoding FLAG-tagged ZNF395 under control of tet-operator elements. The cell line was incubated in doxycycline (Dox) (1µg/ml) for 24h to induce the expression of FLAG-ZNF395. The hypoxic response was produced by injecting N_2_ gas into the incubator to obtain an O_2_ concentration of 2%. Cells were then incubated for 12h and immediately harvested.

### Plasmids

pcDNA3.1-FLAG-ZNF395, pcDNA3.1-FLAG-ZNF395ΔCR1 and pcDNA3.1-FLAG-ZNF395mtCR3 were described previously [23,25]. pcDNA3.1-FLAG-ZNF395mtNES was generated by site-directed mutagenesis. The ISG56-pGL3-Luc construct is described by Grandvaux [30]. Deletions and point mutations were introduced either by cloning appropriate PCR products into pGL3-Luc or by site-directed mutagenesis. IFI44-Luc was obtained by cloning a PCR fragment generated from genomic DNA, encoding 560 nucleotides of the upstream regulatory region including the +1 into pGL3-Luc. The expression vector for IRF3-5D is described by Lin et al. [31]. HA-IKKα (Addgene plasmid 15469), HA-IKKβ (Addgene plasmid 15470) and FLAG-IKKβ, FLAG-IKKβ-K44M, FLAG-IKKβ-S177/181E (Addgene plasmids 11103, 11104, 11105), are published [32,33].

### RT-PCR, microarray

Total RNA was prepared by the RNeasy Mini Kit from Qiagen. cDNA synthesis and hybridization to Affymetrix Exon 1.0 ST array was performed by the group of Prof. Nürnberg (CCG, Cologne, Germany). The raw data were processed using Affymetrix Power Tools, version 1.12.0 and the Robust Multiarray Average (RMA) algorithm [34]. Downstream statistical analysis was carried out using the R language for statistical computing, version 2.10.0. For quantitative real-time PCR, 2µg of RNA were reverse transcribed using random primer and the SuperScript VILO cDNA Synthesis Kit (Invitrogen). Real-time PCRs were performed with SybrGreen and a LightCycler system (Roche Diagnostics). The significance was calculated by the t-test with paired samples. The primer sequences are given in table 1. Our microarray experiment data were deposited on the GEO database (http://www.ncbi.nlm.nih.gov/geo) and assigned the accession number GSE44327.

**Table 1:** Primers used for qRT-PCR.

Gene	Forward primer (5´to 3´)	Reverse primer (5´to 3´)
IFI16	TGCACCCTCCACAAG	CCATGGCTGTGGACATG
IFI44	TGGCAGTGACAACTCGTTTGA	CCGCTTCCCTCCAAAA
ISG56	TCATCAGGTCAAGGATAGTCTG	GGTGTTTCACATAGGCTAGTAG
MEF2C	GCCCTGAGTCTGAGGACAAG	AGTGAGCTGACAGGGTTGCT
PEG10	AACAACAACAACAACTCCAAG	TCTGCACCTGGCTCTGCAG
IFIT2	ACTGCAACCATGAGTGAGAAC	GCCTCGTTTTGCCCTTTGAG
ZNF395	CGAAAAAGAAAGAACTCTGTG	CTGTGTCCCCCAGATGGAG
HPRT	TGACACTGGCAAAACAATGCA	GGTCCTTTTCACCAGCAAGCT

### Small interfering RNA interference

Small interfering RNAs (siRNA, siGenome SMARTpool) were obtained as a pool of four annealed double-stranded RNA (dsRNA) oligonucleotides: IKKα (M-003473-02) and IKKβ (M-003503-02) from Dharmacon, ZNF395 (sc-77820) from Santa Cruz. As a control, siRNA against the oncogene E6 of HPV8 (8E6) [35] was used in the same amounts as the specific siRNAs, respectively. 6 cm-dishes of U87-MG and RTS3b cells were transfected with 150pmol siZNF395, 250 or 500pmol siIKKα, β or the appropriate amount of control siRNA using Lipofectamine RNAiMax (Invitrogen).

### Antibodies, Chromatin Immunoprecipitation (ChIP) Assay, gel shift

The rabbit polyclonal antibody against ZNF395 was described previously [21]. The M2 FLAG antibody was from Sigma-Aldrich, the anti-HA-antibody from Roche, the anti-actin antibody from Santa Cruz, and antibodies against IKKα and IKKβ were from Cell Signaling. Preparation of whole cell extracts and co-immunoprecipitations were conducted as described previously [36]. Dephosphorylation reactions were performed with λ-phosphatase (Santa Cruz) according to the manufacturer’s protocol. ChIP Assay was done according to the ChIP Assay protocol by Upstate Biotechnology. The presence of ISG56 promoter fragments in the precipitate was analyzed by qPCR using the LightCycler system (Roche Diagnostics) and primer flanking the sequences from -173 to +1 of the ISG56 promoter (forward 5´-TGAGATCTGGCTATTCTGTCTTGTGG-3; reverse 5´-ATGGTTGCAGGTCTGCAGTTTATCTG-3´). Gel shifts were performed as described previously [25] with the following ds oligonucleotide (only the upper strand is given): ISRE-wt: 5´-TTTAGTTTCACTTTCCCCTTTCGGTTTCCCTAGGT-3´; ISRE-mut114/101: 5´-TTTAGTGTCACTTTCCCCTGTCGGTTTCCCTAGGT-3´, which were labeled with ^32^P-γ-ATP or used as competitors.

## Results

### ZNF395 activates genes associated with the innate immune response and cancer

**Figure 1:**
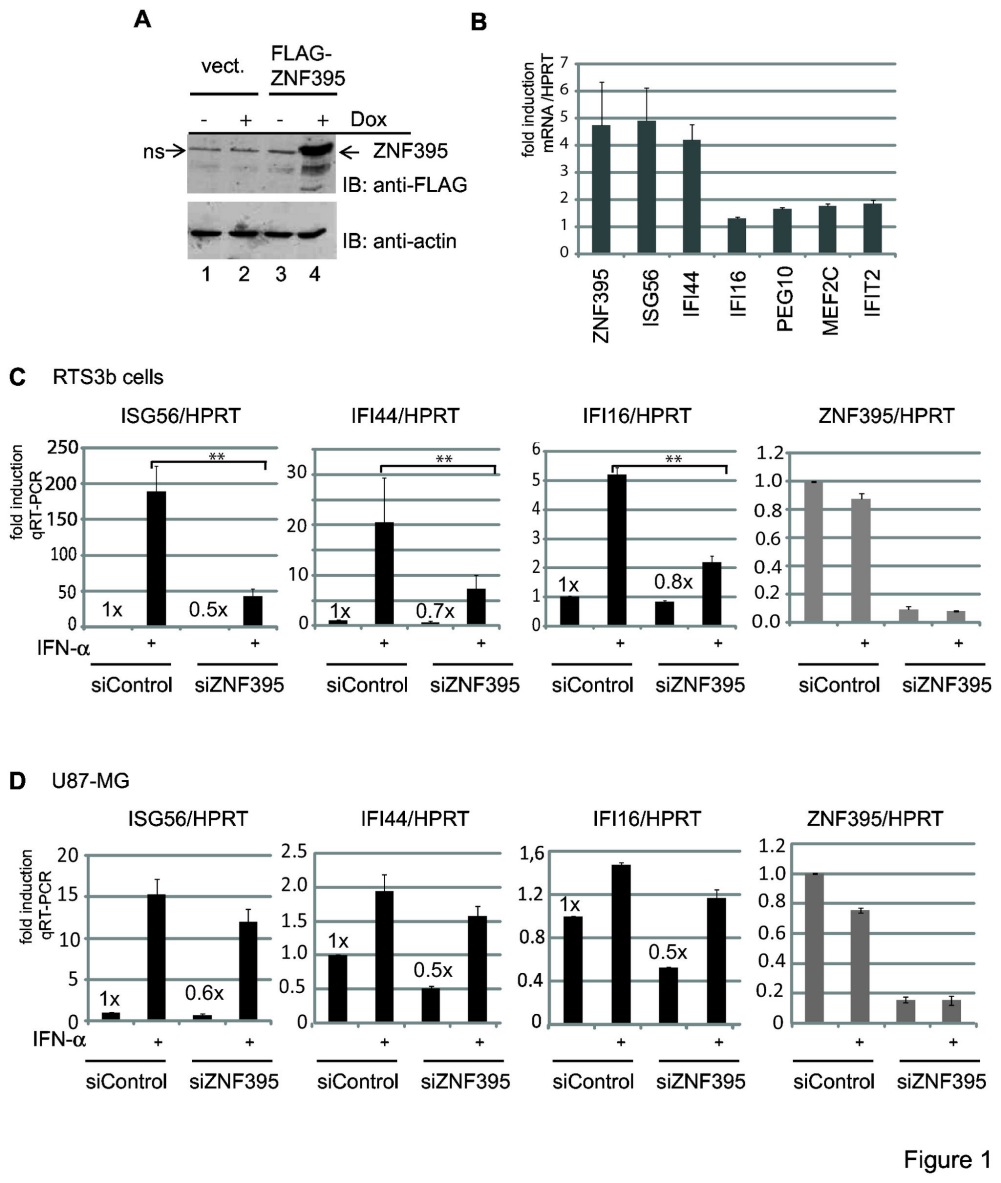
ZNF395 activates innate immune response and cancer-associated genes. (**A**) Stably transfected RTS3b cells expressing the tet repressor and FLAG-tagged ZNF395 under control of the tet inducible promoter (lanes 3, 4) or the empty vector pcDNA4/TO (lanes 1, 2) were either grown in the absence (lanes 1, 3) or presence of Dox (lanes 2, 4) for 24h. Extracts were used for ImmunoBlot (IB) which was developed with the FLAG antibody and an anti-actin antibody as control. ns (non specific band) (**B**) Total RNA isolated from RTS3b TR-FLAG-ZNF395 cells, either grown with or without Dox was used for qRT-PCR to analyze the expression of the factors shown in the graph. The corresponding values were normalized to the values for the housekeeping gene hypoxanthine guanine phosphoribosyl transferase (HPRT) and those obtained from cells grown in the absence of Dox were set as 1 for each factor. The graph represents the means of two independent experiments each performed in duplicate. The error bars represent the standard deviations. (**C**, **D**) RTS3b and U87-MG cells were transfected with control siRNA or siRNA targeting ZNF395 in duplicate. One set of samples was treated with solvent and the other with IFN-α, before total RNA was isolated. QRT-PCR was performed with the specific primer to amplify ISG56, IFI44, IFI16 and ZNF395 transcripts. CP-values obtained for the various factors were normalized against those for the housekeeping gene HPRT. The value with RNA from solvent treated cells transfected with siControl was set as 1 in each case. The fold activations were calculated according to the comparative threshold method described in Pfaffl et al. [64]. QPCRs were performed four times and the standard deviations are given. The values provided in the figure reflect the non-induced basal expression level in the absence of ZNF395 (** p <0.01).

We have isolated the cDNA for ZNF395 from HaCat cells [21]. For this reason, we used a human keratinocyte cell line to investigate the transcriptional events due to the expression of ZNF395 by oligonucleotide microarrays. Since we have observed previously that overexpression of ZNF395 induced cell growth inhibition [23], we generated a cell line, based on the skin cancer derived RTS3b line [27], allowing the inducible expression of ZNF395 controlled by doxycycline (Dox). Five independent cultures were either incubated in the presence or absence of Dox. While a total protein extract was prepared from one sample set to confirm the expression of ZNF395 by ImmunoBlot (IB) (Figure 1A), the residual four sets were used for isolation of total RNA, which was transcribed to cDNA followed by hybridization to the GeneChip human Exon 1.0 ST array from Affymetrix. Gene expression differences were assessed by Students t-test. Genes with a false discovery rate p < 0.05 and a fold change > 1.5 were considered to be differentially expressed. Ten known genes, which are listed in Table 2, passed these criteria. Six of the ZNF395-induced genes are known to be regulated by IFN, which are IFIT1 (ISG56), IFIT2 (ISG54), IFI16, IFI44, CARD6, and SAMD9. The genes MACC1, PEG10, CALCOCO1, and MEF2C have been found to be implicated in cancer (Table 2). ZNF395 mediated activation of IFIT1/ISG56, IFIT2/ISG54, IFI44, IFI16, PEG10, and MEF2C in these cells was confirmed by quantitative (q) RT-PCR with RNA prepared from Dox-treated RTS3b-TR-FLAG-ZNF395 cells compared to RNA from untreated cells (Figure 1B). It is very surprising that we did not detect any gene repressed by ZNF395 although in reporter gene assays ZNF395 efficiently repressed specific promoters [25].

**Table 2:** ZNF395-induced genes, identified by microarray, are involved in the innate immune response and cancer progression.

**Gene name; accession**	**fold ind.**	**t-test**	**full name; function**
**Interferon regulated:**			
IFIT1/ISG56; NM_018660	2.94	0.0087	Interferon-induced protein with tetratricopeptide repeats 1, antiviral activity [38]
IFI16; NM_005531	1.51	0.0278	Interferon-induced protein 16, (Hin200), innate sensor for DNA, induces senescence [43]
IFI44; NM_006417	2.89	0.0280	Interferon-induced protein 44, anti-proliferative and antiviral activity [42]
IFIT2/ISG54; NM_001547	1.6	0.044	Interferon-induced protein with tetratricopeptide repeats 2, induces apoptosis, antiviral activity [60]
CARD6; NM_032587	1.58	0.058	Caspase recruitment domain family, member 6, NF-ĸB activator, associated with carcinomas [61]
SAMD9; NM_017654	1.6	0.066	Sterile alpha motif domain containing 9; innate antiviral factor, TNFα responsive [62]
**Cancer-associated**:			
CALCOCO1; NM_020898	1.67	0.0087	Calcium binding and coiled-coil domain 1, transcriptional coactivator with TCF/LEF and β-catenin [58]
MEF2C; NM_002397	1.64	0.0087	Myocyte enhancer factor 2C, potential oncogene in T cell acute lymphoblastic leukemia [57]
PEG10; NM_015068	1.57	0.0278	Paternally expressed 10, involvement in hepatocellular carcinoma [63]
MACC1; NM_182762	1.59	0.0352	Metastasis associated in colon cancer 1; associated with colon cancer metastasis [59]
**Unknown:**			
ARMCX6; NM_019007	1.66	0.0435	Armadillo repeat containing, X-linked 6; unknown

RNA was purified from four treated and untreated cell samples, respectively, reverse transcribed and hybridized to eight independent oligonucleotide arrays. The average fold change and the FDR q value for Student’s t-test are provided. Genes in which expression was induced more than 1.5 fold with a q < 0.05 are listed, including their accession number and known functions.

The finding that six of the genes identified in the microarray are known regulated IFN genes implied a role of ZNF395 in the IFN-α-mediated stimulation of these target genes. In order to analyze the contribution of ZNF395 we transiently transfected normal RTS3b cells with control siRNA (siControl), which was directed against the HPV8E6 oncogene that is not expressed in these cells, or siRNA targeting ZNF395 (siZNF395) and incubated the cells 40h later with IFN-α for 6h. QRT-PCR revealed that the expression of ZNF395 was not altered in the siControl transfected cells due to IFN-α, indicating that ZNF395 is not regulated by this signal. The ZNF395-specific siRNA reduced the ZNF395 expression more than 90% (Figure 1C). IFN-α induced the expression of ISG56 190-fold in the control cells. Upon the transfection of the siRNA targeting ZNF395, the IFN-α-mediated activation reached only 43-fold. The mRNA level for IFI44 was increased 20-fold by IFN-α, while the suppression of ZNF395 yielded a 7.4-fold induction by IFN-α. We also analyzed the expression of IFI16, which was only weakly induced in the microarray shown in table 2. IFI16 transcripts rose 5-fold in response to IFN-α in siControl transfected cells and in cells where ZNF395 was suppressed, IFN-α activated 2.2-fold (Figure 1C). Thus, the lack of ZNF395 strongly impaired the IFN-α-mediated induction of ISG56, IFI44 and IFI16 in the epithelial RTS3b cells. We confirmed the contribution of ZNF395 in the IFN-α-dependent stimulation of ISG56 and IFI44 expression in the astrocytoma cell line U87-MG. We chose this cell line since Murat et al. reported that ZNF395 is induced upon hypoxia in U87-MG cells [5]. The suppression of ZNF395 by siRNA decreased the basal level of transcripts for ISG56 and IFI44 by about 40 and 50%, respectively, in the unstimulated cells (Figure 1D). The ISG56 expression was induced 15-fold in the control cells and 12-fold in siZNF395-transfected cells by IFN-α, while IFI44 expression was only increased 2-fold due to IFN-α, and 1.6-fold when ZNF395 was knocked down. IFI16 was hardly activated independent whether ZNF395 was present or not. Thus, the overall effects of IFN-α as well as the knockdown of ZNF395 were weaker in U87-MG compared to RTS3b cells. Nevertheless, these results demonstrate that ZNF395 is required for the full IFN-α response to induce the expression of IFI44, ISG56 and IFI16.

### ZNF395 requires its CR3 DNA-binding region and the two ISREs to stimulate the ISG56 promoter

**Figure 2:**
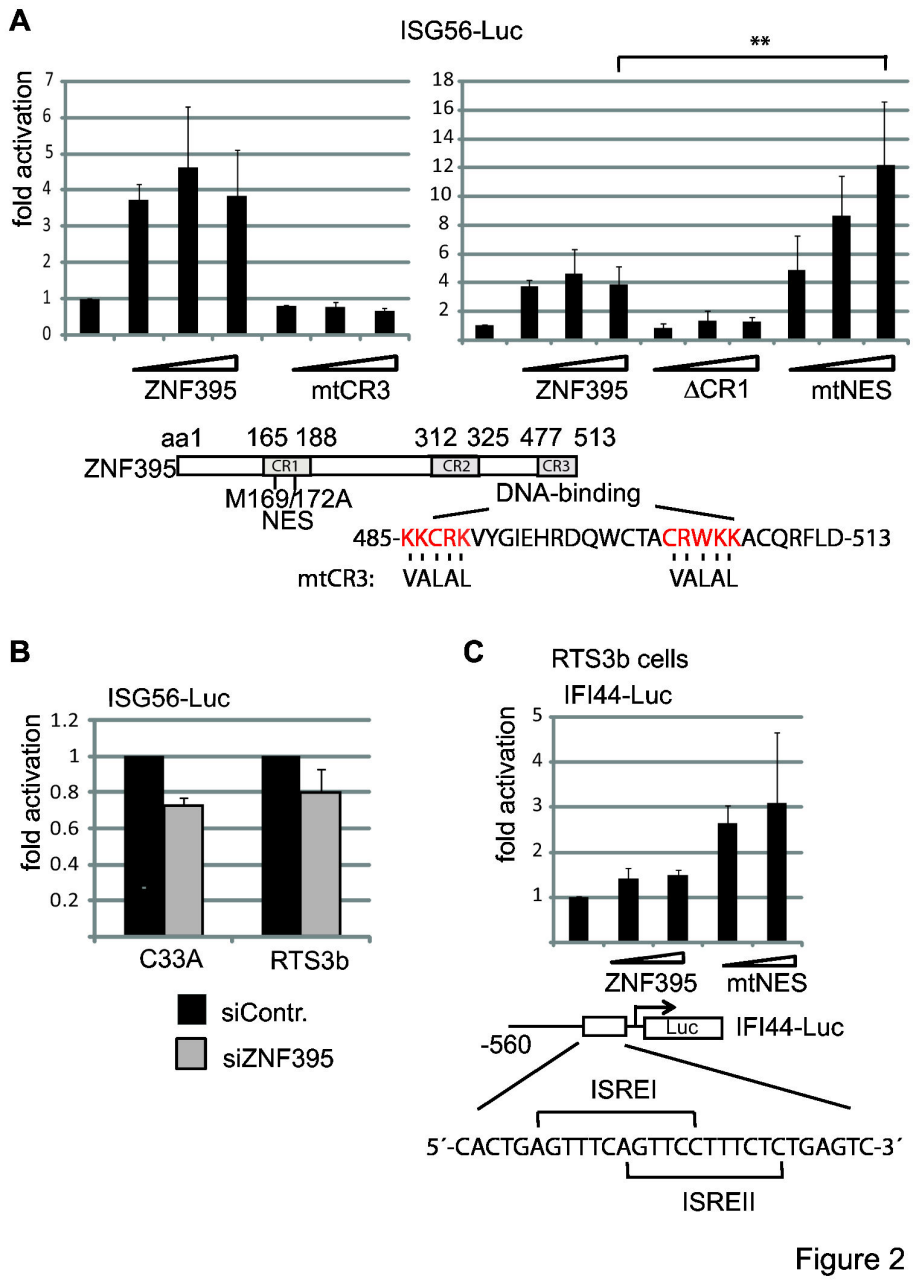
ZNF395 activates the ISG56 promoter and requires its DNA-binding domain and CR1. (**A**) RTS3b cells were seeded in six-well plates and transiently transfected with 500ng of the ISG56-Luc reporter construct and increasing amounts (5, 10, 20ng) of expression vector for FLAG-ZNF395 or the different mutants per well, as indicated. The structure of ZNF395 with its conserved regions CR1, CR2 and CR3 is depicted beneath the graphs including the sequence of the C-terminal 25 amino acids, which are conserved to the E-tail of TCF-1E and TCF-4E [52]. The M at pos. 169 and 172 were changed to A in mtNES while in ΔCR1 the amino acids from 165 to 188 were deleted. The amino acids that were mutated in mtCR3 and leading to loss of DNA-binding are indicated. (**B**) RTS3b and C33A cells were first transfected with siControl or siZNF395 and 24h later with the ISG56-Luc reporter construct. All graphs represent the results of at least three independent experiments. The standard deviations are given. (**C**) RTS3b cells were transiently transfected with the Luciferase reporter construct containing the IFI44-promoter including 560bp bases upstream of the initiation site. The segment harbors two overlapping ISREs, which have been shown to mediate the IFN-dependent induction of IFI44. The expression vector for ZNF395 and ZNF395mtNES were co-transfected as in A.

To investigate the role of ZNF395 in the induction of ISG56, we analyzed recombinant ZNF395 in transient transfection assays with a reporter construct containing 654bp of the upstream control region of ISG56 that includes the promoter. Overexpression of ZNF395 induced the ISG56 promoter 4-fold, which is in the range observed in the microarray (Figure 2A). We identified the regions in ZNF395 required for activation of the ISG56 promoter. The CR3 of ZNF395 is necessary for sequence-specific DNA-binding to a CG-rich element present in HPV8 and in the Huntington disease gene promoter [22,23]. ZNF395mtCR3, with point mutations abolishing DNA binding to the HPV8 promoter [23] lost its ability to activate ISG56-Luc expression. In addition, ZNF395ΔCR1, lacking the CR1 region, failed to activate. Nuclear-cytoplasmic shuttling of ZNF395 was shown to require a nuclear export signal (NES) located within the CR1 [22,23]. In order to differentiate between the role of the NES and the CR1, we introduced two point mutations to destroy the NES. ZNF395mtNES activated the ISG56 promoter up to 10-fold (Figure 2A). Thus, residues present within CR1 are engaged in ZNF395-mediated activation of transcription which was not further addressed here. The higher activation of ZNF395mtNES correlated with the exclusive nuclear localization of ZNF395mtNES (unpublished results) [22]. The expression of the mutated proteins has previously been shown [23,25]. To confirm these activations, we revealed an impact of endogenous ZNF395 on the promoter activity obtained with ISG56-Luc by transiently co-transfecting siZNF395 or siControl with the ISG56 Luc reporter construct. The suppression of ZNF395 resulted in a 20% reduction of the Luc activity in RTS3b and C33A cells, respectively (Figure 2B). Ectopically expressed ZNF395 was also able to activate the IFI44 promoter present in a reporter construct. This included 590bp upstream of the initiation site with two overlapping ISREs from pos. -46 to -64 that were mapped previously to mediate the induction by IFN-α and IFN-β [37]. Again, ZNF395mtNES showed increased activation, although the total effects were smaller compared to ISG56-Luc (Figure 2C).

**Figure 3:**
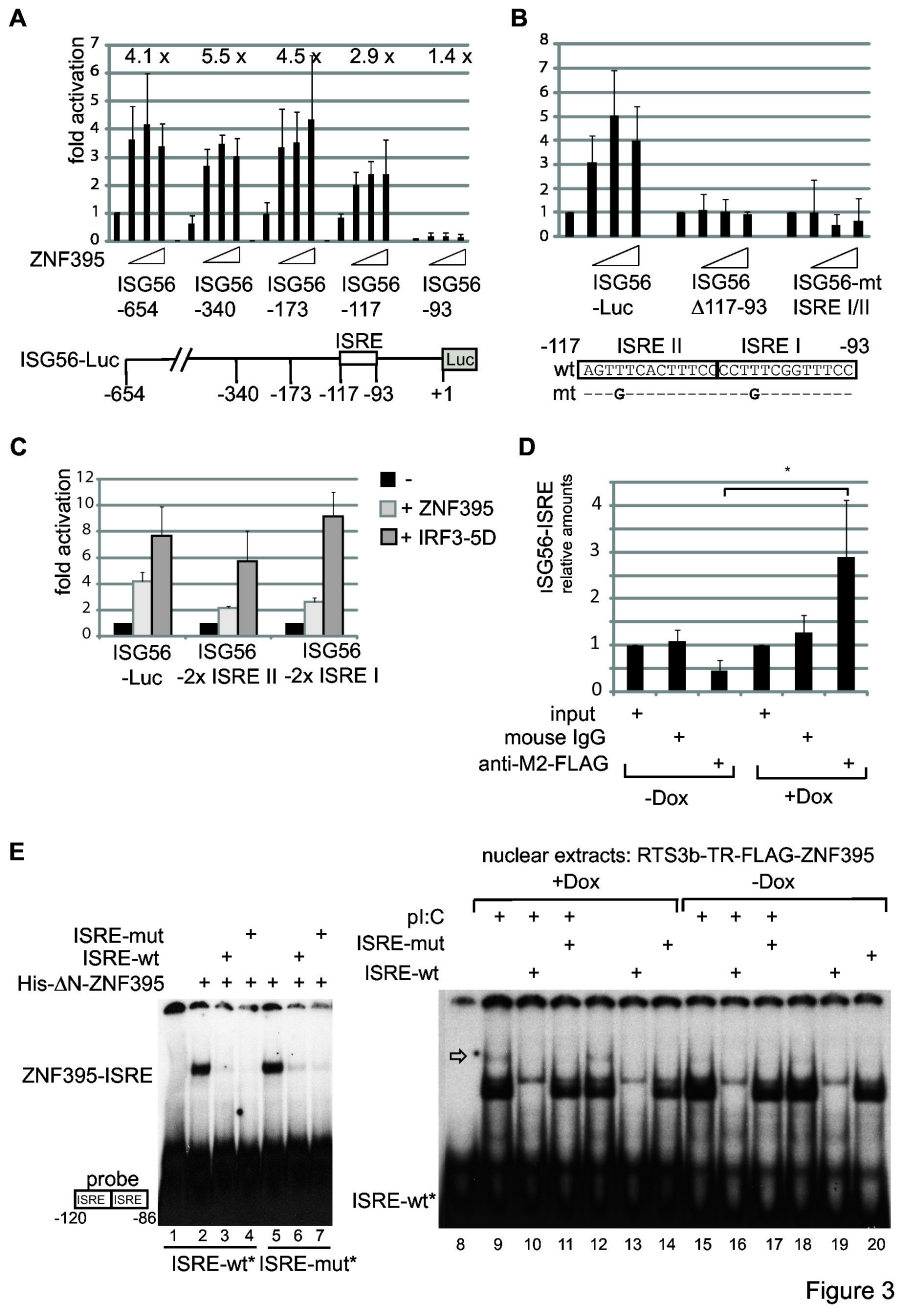
ZNF395 acts through ISREI and ISREII to stimulate the ISG56 promoter. (**A**) Sequential deletions starting from the 5´ end of the ISG56 upstream regulatory region were introduced into the ISG56-Luc construct as indicated in the figure. The corresponding luciferase reporter constructs were co-transfected with 5, 10 and 20ng of the FLAG-ZNF395 expression vector. The relative luciferase activity of the full length ISG56-Luc (=ISG56-654) construct was set as 1. The numbers above each set represent the fold activations induced by ZNF395 with the relative activity of each mutated reporter construct set as 1. (**B**) The construct ISG56Δ117-93 contains a deletion of the two ISREs within the context of the full length ISG56-Luc construct which harbors the upstream region up to -654bp while in ISG56-mtISRE I/II a T in each ISRE has been changed into G, as indicated beneath the graph. All reporter constructs were co-transfected again with 5, 10 or 20ng of the expression vector for FLAG-ZNF395. The sequence of the two ISREs present in the ISG56 promoter with the point mutations that have been introduced is provided. (**C**) Transient transfections with ISG56-Luc reporter constructs that were modified to contain either two copies of ISRE I (ISG56-2x ISRE I) or two copies of ISRE II (ISG56-2x ISRE II) and 5ng of expression vector for ZNF395 or IRF3-5D, respectively. (**D**) ChIP-assay. RTS3b-TR-FLAG-ZNF395 cells were grown in the absence or presence of Dox, cross-linked and subjected to a ChIP assay with antibody against the FLAG-tag and control mouse IgG. The precipitated DNA segments were amplified with a LightCycler using primers flanking the ISREs of the ISG56 promoter. The ISG56-Luc reporter construct was included as standard to allow a quantification. The copy number obtained for the input was set as one for each extract and the fold enrichment was calculated. The PCRs were performed in triplicate (* p=0.05). (**E**) Gel shift analysis. Bacterially expressed his-tagged purified ΔN-ZNF395 (lacking amino acids 1-114) was incubated with 200pg ISREI-wt (lanes 1-4) or ISRE-mut oligonucleotide (lanes 5-7), both radioactively labeled with ^32^P-γ-ATP and the binding was analyzed with a native PAA gel. In lanes 3 and 6, a 250-fold excess of unlabeled ISRE-wt and in lanes 4 and 7, of ISRE-mut oligonucleotide were added as competitors. In the gel shift shown on the right, nuclear extracts prepared from RTS3b-TR-FLAG-ZNF395 cells, either incubated in the absence or presence of Dox and polyI:C, as indicated, were incubated with labeled ISRE-wt oligonucleotide and a 250-fold excess of unlabeled ISRE-wt or ISRE-mut oligonucleotide. The position of the putative ZNF395-ISRE complex is indicated by an arrow.

We then determined the sequence requirements of ZNF395 in the ISG56 promoter. As shown in Figure 3A, although it slightly reduces activity, removal of the sequences up to -117 did not eliminate the activation by ZNF395. The deletion of the segment from -117 to -93 dramatically decreased the basal activity of the promoter, and eliminated any stimulation by overexpressed ZNF395. This DNA segment harbors the IFN-stimulated-response elements (ISRE) I and ISREII that mediate induction of ISG56 by IRF3 and the IFN-α sensitive transcription factor ISGF3 [38]. Two point mutations within the ISREs, which were shown to eliminate the responsiveness to IRF3 [30] and to IFN-α (data not shown) abolished activation by overexpression of ZNF395 (Figure 3B), demonstrating that ZNF395 acts through either one or both ISRE elements. With two modified constructs containing either two copies of the ISREII (ISG56-2x ISREII) or two copies of the ISREI (ISG56-2x ISREI), ZNF395 obtained half of the activation compared to the wt promoter. The modifications did not significantly affect IRF3-5D, a constitutive active mutant of IRF3 [31], in its capacity to increase luciferase-activity (Figure 3C). Thus, the sequence composition of the ISREI and -II present in the wt promoter is optimal for ZNF395 to activate. To test whether ZNF395 is located at the endogenous ISG56 promoter, a ChIP assay with RTS3b-TR-FLAG-ZNF395 cells was performed. The amount of the fragment containing the ISG56-specific ISREs which was pulled-down by the FLAG-antibody from the extracts was determined by qPCR. With extracts from cells grown in the presence of Dox to induce the expression of FLAG-ZNF395, the FLAG antibody precipitated 288% of the input (+ Dox, Figure 3D), representing a 2.3-fold increase compared to the IgG control which recovered 127% of the input. To confirm that ZNF395 can directly bind to the ISREs, we performed gel shifts. His-tagged purified ZNF395 shifted a radiolabeled oligonucleotide encoding the two ISREs of the ISG56 promoter (ISRE-wt). Surprisingly, the oligonucleotide encoding these two ISREs with the mutations T-G in pos. 114 and 101 (ISRE-mut) was equally well bound and was able to compete for the binding when added in excess (Figure 3E). The direct binding of ZNF395 was confirmed with nuclear extracts prepared from the RTS3b-TR-FLAG-ZNF395 cells either grown with or without Dox and polyI:C, a mimic of dsRNA, respectively. One prominent band was visible with all extracts, independent of polyI:C and Dox. The homologues ISG56-ISRE-wt oligonucleotide, but not the mutated ISRE-mut oligonucleotide was able to compete this complex. Upon induction of ZNF395 expression by Dox, an additional faint band migrating just above the major complex appeared (Figure 3E, lanes 9, 12). This complex disappeared in the presence of an excess of unlabeled ISRE-wt as well as ISRE-mut oligonucleotide. Since this band only was detected with extracts of cells overexpressing recombinant ZNF395 and behaved like purified ZNF395 regarding the sequence specificity, it might represent ZNF395 bound to the ISREs. These observations imply that ZNF395 is directly located at the ISREs. However, the sequence specificity observed with the transient transfections may not rely on ZNF395 binding.

### IKK marks ZNF395 for proteasomal degradation

The results described so far show that ZNF395 is required for the full induction of ISG56, IFI44 and IFI16. These IFN-stimulated factors are known to be part of an antiviral innate immune response. In addition, ISG56 can be directly activated by IRF3 upon TLR3 signaling in response to dsRNA. TLR3 is expressed in cells of the immune system and epithelial cells, as are RTS3b (data not shown).

**Figure 4:**
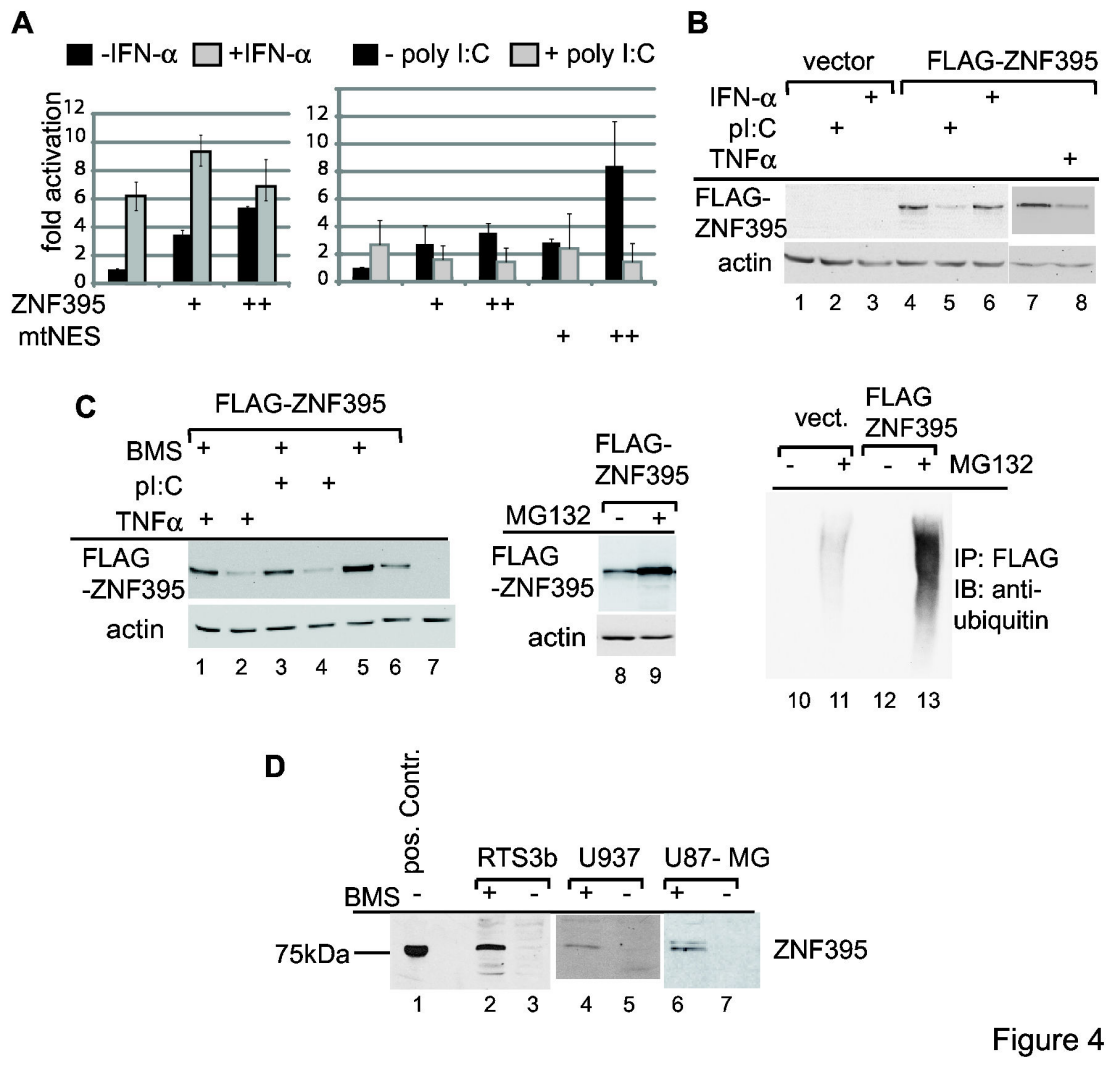
Active IKK marks ZNF395 for degradation. (**A**) RTS3b cells were transiently transfected with 5ng (+) and 10ng (++) of the expression vector for FLAG-ZNF395 and in the experiments shown in the right graph, 5ng (+) or 10ng (++) of the vector for ZNF395mtNES was included. The transfected cells were treated either with IFN-α (left graph) or with polyI:C (right graph) as indicated. The bars represent the fold activations calculated from three independent experiments and the standard deviations are included. (**B**) Cells were transiently transfected with expression vector for FLAG-ZNF395 (lanes 4-8) or the empty vector (lanes 1-3) and treated either with polyI:C (lanes 2, 5), IFN-α (lanes 3, 6), TNFα (lane 8) or solvent (lanes 1, 4, 7). An IB with the anti-ZNF395 antibody and the anti-actin antibody was performed. (**C**) Cells transiently transfected with the FLAG-ZNF395 vector or the empty vector (in lanes 7, 10, 11) were treated with TNFα (lanes 1, 2), poly I:C (lanes 3, 4) or MG132 (lanes 9, 11, 13). BMS-345541 was added to the cells used in lanes 1, 3, 5 and the solvent DMSO in lanes 2, 4, 6, 7, 8. The analysis of ZNF395 expression was done by IB using the anti-FLAG and anti-actin antibody. In lanes 10–13, FLAG-ZNF395 was precipitated by M2-FLAG-agarose and the IB was performed with an antibody against ubiquitin. (**D**) RTS3b (lanes 2, 3), U937 (lanes 4, 5) or U87-MG cells (lanes 6, 7) were incubated in medium containing BMS-345541 (+) or DMSO (-) and analyzed for ZNF395 expression by an IB developed with the anti-ZNF395 antibody. In lane 1, extracts prepared from RTS3b TR-FLAG-ZNF395 cells used in Figure 1 were used as a control.

We wanted to analyze the fate of ZNF395 within this pathway by transient transfections. Incubating transfected cells with IFN-α increased the ISG56 promoter in the ISG56-Luc construct by 6-fold, which was not affected by the co-expression of ZNF395 (Figure 4A). When we incubated transiently transfected cells with polyI:C, to stimulate signaling by TLR3, we observed a 3-fold induction of the ISG56 promoter in average. However, polyI:C reduced activation obtained by overexpressed ZNF395 by 50%. This was even more obvious with ZNF395mtNES, whose 8.3-fold activation decreased to 2.3-fold due to polyI:C, representing a 70% repression (Figure 4A, right graph). An IB with extracts from transiently transfected cells revealed that the amount of recombinant ZNF395 was markedly reduced upon incubating the cells with polyI:C. This was not observed when IFN-α was added to the cell culture medium (Figure 4B). Similar to ligand-bound TLR3, the pro-inflammatory cytokine TNFα results in the activation of the canonical IKK pathway [12]. Treatment of transfected cells with TNFα decreased the protein level of recombinant ZNF395 as well (Figure 4B, compare lanes 7 and 8). Thus, activation of IKK signaling may target recombinant ZNF395 to degradation. To address a role of IKK in the control of the stability of ZNF395 we used BMS-345541, a highly specific inhibitor of IKKα and β [39,40]. The IKK inhibitor increased the amount of recombinant FLAG-ZNF395 and eliminated the polyI:C- and TNFα-mediated degradation of FLAG-ZNF395, respectively (Figure 4C). Inhibition of the proteasome by the inhibitor MG132 led to a higher amount of recombinant ZNF395 (Figure 4C, lanes 8, 9) which supports the notion that ZNF395 is constantly degraded in the cell. An IB analyzing FLAG-ZNF395 precipitated from extracts of transiently transfected cells that were treated with MG132 indicated that FLAG-ZNF395 was highly ubiquitinated in the presence of MG132 (Figure 4C, compare lanes 11 and 13). Also endogenous ZNF395 expressed in proliferating cells was stabilized by BMS-345541 since endogenous ZNF395 was only detectable in extracts from RTS3b, U937 and U87-MG cells, when the cells were incubated with BMS-345541 for 24h (Figure 4D).

**Figure 5:**
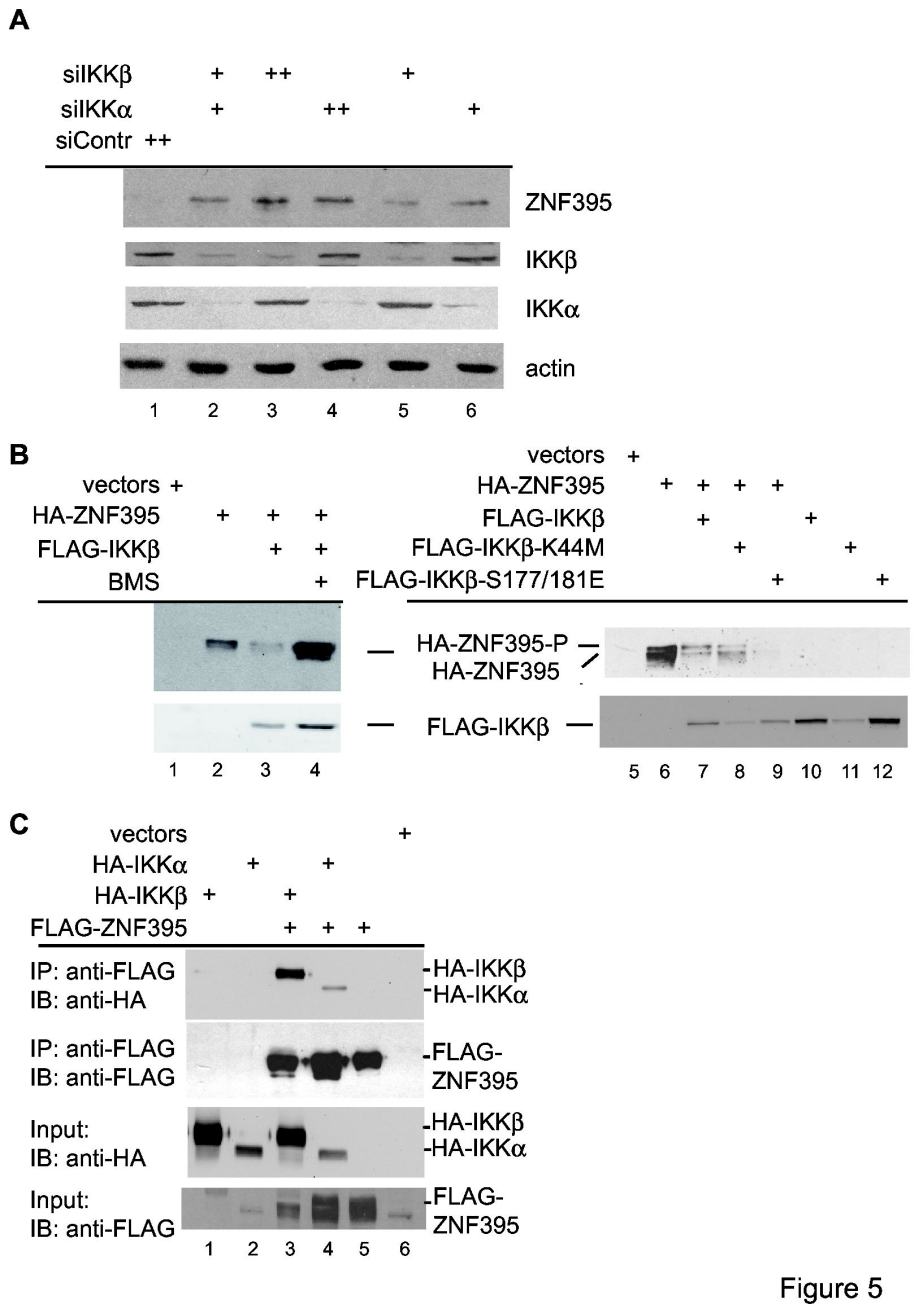
IKK targets ZNF395 to degradation. (**A**) Analysis of extracts of U87-MG cells that were transfected with 500pmol control siRNA (siContr) (lane 1), with a mixture of 250pmol of siIKKα and siIKKβ each (lane 2), with 500 (++) pmol siIKKβ or siIKKα (lanes 3 and 4), and with 250pmol (+) siIKKβ (lane 5) or siIKKα (lane 6) (in both cases 250pmol siContr was included to keep the total amount of siRNA constant). Total cell extracts were prepared 48h later and analyzed for the expression of ZNF395, IKKα, IKKβ and actin with specific antibodies by IB. (**B**) In lanes 1-4, extracts of RTS3b cells that were transiently transfected with expression vectors for HA-ZNF395 and FLAG-IKKβ were used. Where indicated, BMS-345541 was added to the cells 24h prior to harvesting. In lanes 5-12, the vector for HA-tagged ZNF395 or the empty vector was co-transfected with the plasmids encoding wt FLAG-IKKβ, FLAG-IKKβ-K44M, a kinase inactive mutant, FLAG-IKKβ-S177/181E, a constitutive active mutant, as indicated. ZNF395 expression was monitored in an IB developed with the anti-HA-antibody, while the expression of IKKβ proteins was detected by the FLAG antibody. (**C**) Co-immunoprecipitation. C33A cells were transfected with expression vectors for FLAG-ZNF395, HA-IKKα or HA-IKKβ in various combinations, as indicated. Immunoprecipitations were performed with M2-FLAG agarose and bound proteins were detected by an IB developed with the anti-HA and anti-FLAG antibody. 50µg of total extracts were used as input control to monitor the presence of ZNF395, IKKα and IKKβ in the extracts used for the co-immunoprecipitation, shown in the two blots at the bottom.

We confirmed the role of IKK in controlling the stability of ZNF395 by siRNA-mediated knockdown. Transfecting U87-MG cells with increasing amounts of siRNAs targeting either IKKα or IKKβ, resulted in the dose-dependent detection of endogenous ZNF395. This was not observed with the control siRNA, which was used in the highest amounts (Figure 5A, compare lane 1 with 3, 5 and 4, 6). Thus, both endogenous IKKα and IKKβ are required to induce the proteolytical degradation of ZNF395. We also analyzed the effect of IKKβ overexpression on recombinant ZNF395. The IB shown in Figure 5B revealed that co-expression of IKKβ enhanced the degradation of recombinant ZNF395 which could be inhibited by BMS-345541 (lanes 2-4). Co-expression of the kinase inactive mutant of IKKβ-K44M led to the accumulation of a faster migrating form of FLAG-ZNF395 (Figure 5B, lane 8) which may correspond to a less phosphorylated form. The amount of ZNF395 was still reduced upon co-expression of IKKβ-K44M, which may rely on the fact that the inactive mutant acts as a competitor with the wild-type protein and is thus less efficient compared to BMS-345541, which efficiently targets each IKK molecule. In addition, IKKβ-K44M was expressed to rather low amounts (Figure 5B, lane 8). Co-expression of the constitutively active IKKβ-S177/181E eliminated detectable recombinant FLAG-ZNF395 (Figure 5B, lane 9), confirming the notion that active IKK marks ZNF395 for proteasomal degradation. Finally, we show that ZNF395 interacts with both kinases. The FLAG antibody was able to co-precipitate IKKα as well as IKKβ from extracts of cells that have been co-transfected with expression vectors for FLAG-ZNF395 and HA-IKKα or HA-IKKβ, and not when the cells have been transfected with the FLAG-ZNF395 and the empty HA-tag vectors, as shown in Figure 5C.

### Active IKK is required to allow activation of transcription by ZNF395

**Figure 6:**
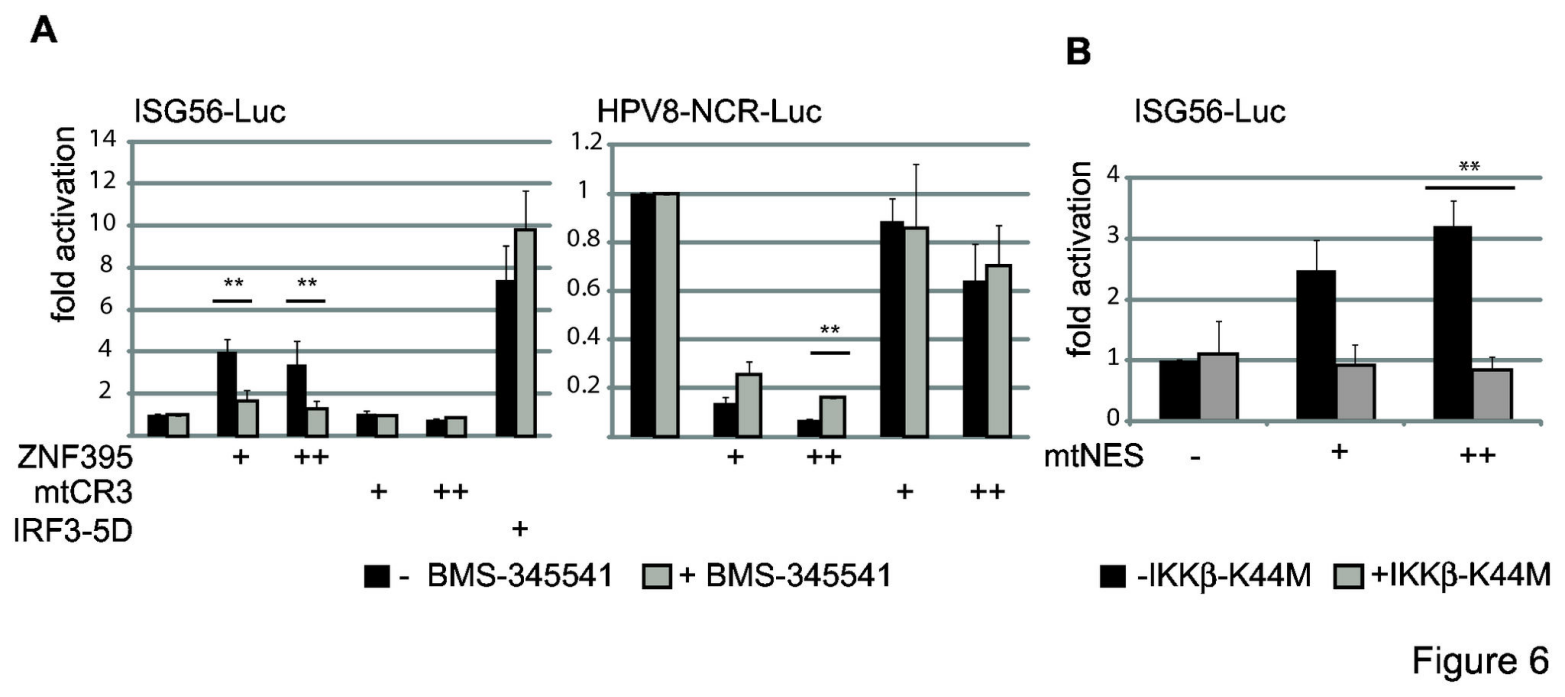
ZNF395 requires active IKK to stimulate the ISG56-promoter. (**A**) RTS3b cells were transfected with the ISG56-Luc reporter construct (left graph) or the HPV8-NCR-Luc reporter construct containing the regulatory region of HPV8 [25] (right graph) and 5 or 10ng of expression vector for ZNF395 or ZNF395mtCR3 in duplicate. In the case of ISG56-Luc, 5ng of expression vector for IRF3-5D was included as control. One set of samples was incubated in the presence of BMS-345541 for 24 h prior to harvesting and analysis. (**B**) RTS3b cells were transiently transfected with the ISG56-Luc reporter-construct and expression vectors for ZNF395mtNES (10, 20ng) and 30ng of the expression vector for IKKβ-K44M or the empty vector. The reduced activation by ZNF395mtNES compared to the experiment shown in Figure 2 may rely on the higher amounts of expression vectors transfected. All graphs represent the fold induction calculated from three independent experiments and include the error bar. The differences that reach significance are labeled with **.

The impact of IKK signaling on ZNF395-mediated activation of the ISG56 promoter was analyzed by treating transiently transfected cells with BMS-345541. Although the concentration of the protein was increased in the presence of the inhibitor, as revealed in Figure 4C, ZNF395 was clearly reduced in its capacity to activate ISG56 expression, as shown in Figure 6A. This was not due to non-specific effects of the inhibitor on transcription, since the activation by IRF3-5D was not affected by BMS-345541. As observed previously, ZNF395 strongly repressed HPV8 transcription, which was largely relieved by mtCR3 [25]. This repression was barely affected by BMS-345541 (Figure 6A). BMS-345541 had no influence on the subcellular distribution of ZNF395 as judged by an immunofluorescence test of RTS3b cells incubated with BMS-345541 (data not shown). The effect of IKK on transcription by ZNF395 was confirmed by the co-expression of the inactive mutant IKKβ-K44M, which eliminated any detectable activation by ZNF395mtNES (Figure 6B). These results demonstrate that signaling by IKK enables ZNF395 to activate transcription and simultaneously accelerates its proteasomal degradation.

### Hypoxia induces the expression of ZNF395, which is transcriptionally active

**Figure 7:**
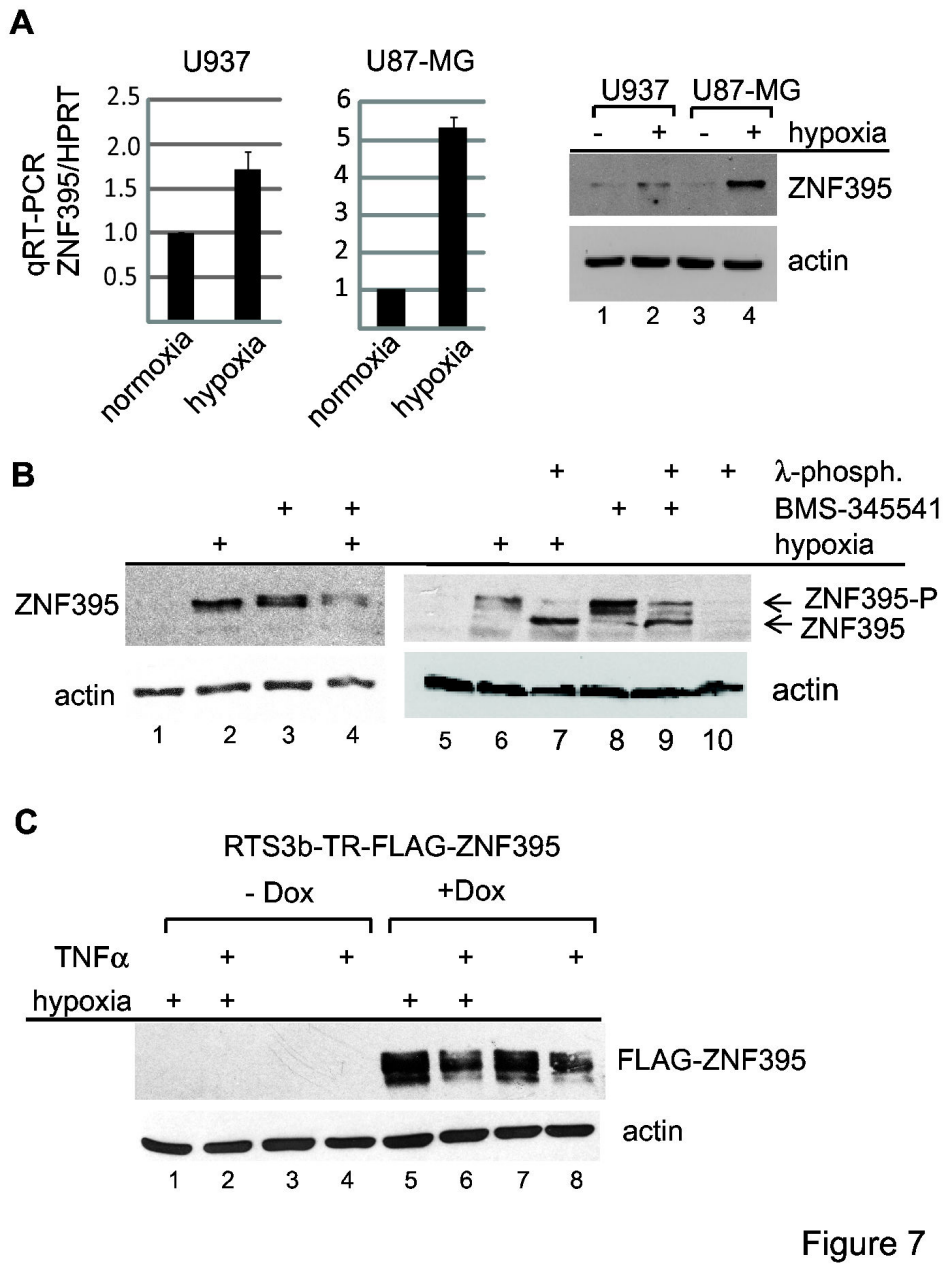
ZNF395 induced during hypoxia is modified by IKK. (**A**) U937 and U87-MG cells were either kept under normoxic or under hypoxic conditions (2% O_2_ atmosphere) for 12h before preparing total cell extracts or RNA. RNA was used for qRT-PCR to analyze the expression of ZNF395, which was normalized to the expression of HPRT. The fold induction was calculated according the comparative threshold cycle [64]. 50µg of protein extracts were used for an IB with the anti-ZNF395. Actin served as the loading control. (**B**) U87-MG cells were grown in ambient or 2% O_2_ atmosphere and either treated with BMS-345541 or left untreated. Fifty µg of total cell extracts were used in an IB to detect ZNF395. In lanes 7, 9 and 10, these extracts were incubated with λ-phosphatase, as indicated. (**C**) 15µg of extracts from RTS3b-TR-FLAG-ZNF395 cells grown in the absence or presence of Dox, hypoxia and TNFα, as indicated in the figure, were analyzed in a IB for the expression of ZNF395, and actin. α.

We considered analyzing the phosphorylation status of endogenous ZNF395, but we were unable to detect endogenous ZNF395 in various cell lines, which may be due to the IKK-mediated degradation of the protein. As already mentioned, data from the literature suggested that ZNF395 is a hypoxia-induced gene [9,41]. We performed qRT-PCR with RNA from U87-MG and U937 cells, which were grown for 12h in 2% O_2_ atmosphere, and found a 5.3- and 1.7-fold increase of ZNF395 expression due to hypoxia, respectively. In correlation, endogenous ZNF395 became detectable by IB with protein extracts from these cells when they were incubated in 2% O_2_ atmosphere (Figure 7A).

To address a post-translational modification of endogenous ZNF395 by IKK, we tested whether BMS-345541, i.e. inhibition of IKK affects the migration of endogenous ZNF395 induced by hypoxia. U87-MG cells were incubated for 12h at 2% O_2_ atmosphere in the presence or absence of BMS-345541. As shown in Figure 7B, upon hypoxia, ZNF395 was detectable with one prominent band. In cells grown in the presence of BMS-345541, independent of hypoxia, the protein appeared with two bands, indicative for a modification of ZNF395 by IKK. ZNF395 induced by hypoxia was highly phosphorylated since it migrated considerably faster after incubating the extracts with λ-phosphatase (Figure 7B, lane 7). Also, a fraction of ZNF395 induced by BMS-345541 was still phosphorylated as demonstrated by λ-phosphatase (Figure 7B, lane 9). Thus, in addition to IKK, ZNF395 seems to be phosphorylated by other kinases. For instance, we have shown previously that ZNF395 is a substrate of the AKT-kinase in the presence of growth factors [23]. It was also obvious that a fraction of ZNF395 stabilized by BMS-345541was not converted to a faster migrating form due to treatment with λ-phosphatase (Figure 7B, lane 9), which was observed in several experiments. It may be possible that ZNF395 undergoes other modifications in the absence of IKK-dependent phosphorylation. To investigate whether hypoxically-induced ZNF395 is resistant to IKK-mediated proteasomal degradation, we used the RTS3b-TR-FLAG-ZNF395 cell line to induce the expression of ZNF395 by Dox. Thus, we can compare the level of ZNF395 independent of its induction by hypoxia. TNFα reduced the amount of FLAG-ZNF395 in extracts of cells that were kept under normoxia and hypoxia, respectively, indicating that ZNF395 is still degradable in response to the activation of IKK in the presence of hypoxia. Transient transfections revealed that ZNF395 stimulates the ISG56 promoter to the same extent, regardless of whether the cells are grown under normoxia or hypoxia (data not shown). These data demonstrate that higher amounts of ZNF395 in the presence of hypoxia rely on the increased promoter activity and are not due to resistance to IKK-mediated accelerated proteolytic degradation. Induction of ZNF395 by hypoxia might thus give rise to a transcriptional active protein.

## Discussion

A gene expression array revealed that the genes whose expression was induced by the hypoxia-inducible factor ZNF395 are part of pathways involved in cancer and in the innate immune response. SiRNA-based knockdown confirmed that endogenous ZNF395 expressed in U87-MG cells and in the keratinocyte cell line RTS3b contributes to the basal transcription of ISG56 and IFI44, since their expression declines in the presence of siRNA targeting ZNF395 in contrast to the control siRNA, confirming the specificity. Most importantly, knockdown of ZNF395 considerably impaired the IFN-α-mediated stimulation of ISG56, IFI44 and IFI16 in the keratinocyte cell line. Although the overall effects, i.e. the induction by IFN-α and impairment due to loss of ZNF395, were less dramatic in U87-MG cells, our results strongly support the notion that ZNF395 is a novel factor modulating the activation of these factors within the first innate immune response upon virus infection. It is well known that ISG56 contributes to establish an antiviral state via multiple effects on viral and cellular functions such as inhibition of translation, viral replication and cell proliferation [38]. IFI44 was shown to have antiviral activity against HCV as well as anti-proliferative activities [42]. IFI16 acts as an intracellular sensor of dsDNA, including viral DNAs to induce an innate immune response [43,44]. A role of ZNF395 in the innate immune response against virus infections is supported by several reports. Genome-wide screens found transcripts for ZNF395 reduced in CD8^+^ T-cells from HIV viremic patients compared to CD8^+^ T lymphocytes from uninfected or HIV-infected therapy-naïve long-term non-progressors. Similarly, ZNF395 expression was downregulated in CD8^+^ T lymphocytes in the acute phase of HCMV infection compared to naïve CD8^+^ T-cells [45,46,47]. Thus, CMV or HIV viral replication might be more efficient at low ZNF395 concentration. A recent study showed that IFI16 acts as a restriction factor for HCMV replication [48]. According to our data, low levels of ZNF395 will result in a reduced IFN-α-dependent induction of IFI16, which will then be favorable for HCMV replication. A co-upregulation of ZNF395 as well as genes involved in the antiviral defense, including the here identified IFN-α-responsive target genes IFIT1/ISG56, IFI44 and IFI16, was found in CD4+ cells isolated from HIV-1-infected individuals. Elevated expression of these factors correlated with high levels of viral load in this study. It was suggested that IFN activation and elevated ISG expression is associated with disease progression in retroviral pathogenesis [49].

We show that ZNF395 requires its DNA-binding domain within the CR3 and both ISREs to activate the ISG56 promoter. An involvement of ISREs in ZNF395-mediated control of gene expression is further supported by the finding that six target genes of ZNF395 identified by the microarray are known IFN-regulated genes with one or more ISREs within their promoter region. Gel shifts with purified proteins and nuclear extracts as well as a ChIP assay support the notion that ZNF395 is present at the ISREs of the ISG56 promoter. Our DNA-binding study in combination with the reporter gene assays indicate that ZNF395 is not responsible for the sequence specificity mediating the activation since ZNF395 bound to the mutated ISREs in vitro, but failed to activate the ISG56-Luc reporter with these mutations. Thus, currently, it is unclear how ZNF395 will increase the expression of these genes. We speculate that ZNF395 may modulate the activity or DNA binding of another cellular factor, i.e. ISGF3, which confers the sequence specificity. The CR3 of ZNF395 encodes a “C-clamp” that is also present in the E-tail isoforms of the wingless/Wnt signaling effectors, TCF1E, TCF4E and the drosophila TCF/pangolin. In the human TCF E-tail isoforms, the C-clamp acts as an auxiliary DNA-binding domain that facilitates recognition of composite DNA sequence motifs composed of the Wnt response element and an RCCG-motif. Thus, the C-clamp is responsible for selective Wnt target gene recognition containing an RCGG-motif [50] and the C-clamp was shown to mediate the cell-type specific and context-dependent Wnt/β-catenin response by TCF1E and TCF4E [51,52]. In contrast to the TCF E-tail isoforms, our previous observations suggest that the C-clamp within the CR3 of ZNF395 is the only motif required for DNA binding since the mutation in CR3 abolished the binding of ZNF395 to the HPV8 promoter in vitro [25]. Since only selective IFN-regulated genes were identified as targets, ZNF395 may recognize a distinct sequence context and/or surrounding sequence in addition to the ISRE motif. Furthermore, the sequence context even seems to specify the effect of ZNF395 on the target promoter. In the experiment shown in Figure 6, overexpression of ZNF395 repressed the HPV8 promoter, but activated the ISG56 promoter in the same cell line. These findings prove that ZNF395 can lead to activation or repression, depending on the promoter and imply the involvement of a co-factor. We have previously shown that the repression of the HPV8 promoter by ZNF395 involves the recruitment of the SIN3A/HDAC complex via direct interaction with its subunit SAP30 [25]. It is even conceivable that ZNF395-dependent recruitment of this complex is involved in the activation of ISG56 since it has been reported that the induction of ISG expression, including ISG56, requires protein deacetylase activity [53].

We provide evidence that active IKK results in the proteasomal degradation of ZNF395. Our finding that ZNF395 directly interacted with IKKα and IKKβ implies that ZNF395 is associated with the IKK complex. The amino acids from position 210-215 of ZNF395 encode the sequence DSGSST, which is homologue to `DpSGΨXpS/T´ (Ψ is a hydrophobic residue) that functions as an IKK phosphorylation motif and binding site for the ubiquitin ligase β-Trcp in some IKK substrates such as CBP, IĸBα, p105, p100, β-catenin and FOXO3a [12]. We are currently testing whether ZNF395 is directly targeted by IKK.

Our data suggest that IKK-activity is required for ZNF395 to activate transcription and directs ZNF395 to ubiquitin-mediated degradation. The IKK-induced turnover of ZNF395 may contribute to promoter clearance to allow reinitiation of transcription. A similar regulation has been described for IRF3. IRF3 undergoes virus-dependent phosphorylation mediated by IKKε/TBK1, which results in the nuclear translocation, DNA binding and increased transcriptional activation. Phosphorylation of IRF3 in response to virus infections also provides a signal for proteasomal degradation [31]. Moreover, proteasomal degradation of both ZNF395 and IRF3 upon activation of an anti-viral response may also dampen the immediate early immune response and thus represent a negative regulation.

Our results illustrate that the expression of ZNF395 is not affected in response to IFN-α, but is induced by hypoxia. Correlatively, ZNF395 was found to be upregulated as part of a response to hypoxia in glioblastomas and neuroblastomas [5,6] and in adipocytes [54]. ZNF395 may be a direct target gene of HIF since its expression was modulated by overexpression or knockdown of HIF-1α [7,55,56], and a ChIP-on-chip analysis identified two sites several kb upstream of the ZNF395 promoter bound by HIF-1α and HIF-2α [41]. We show that hypoxia does not affect IKK-mediated degradation of ZNF395, implying that ZNF395 induced by hypoxia is transcriptionally active.

The results of the microarray also revealed an increased expression of four genes known to be associated with cancer upon induction of ZNF395. These include the transcription factor MEF2C, found to be activated in a subset of T-acute lymphoblastic leukemia cell lines [57], CALCOCO1, that can act as a transcriptional co-activator for the androgen receptor and TCF/LEF in cooperation with β-catenin [58], or MACC1, which is a key regulator of hepatocyte growth factor receptor signaling and was shown to predict colon cancer metastasis [59]. Although further analysis is required to determine a functional role of ZNF395 in controlling the expression of these cancer-associated genes, it is possible that ZNF395 may also affect cancer growth by elevating the expression of these genes in particular under conditions of hypoxia.

Taken together, we show that ZNF395 is required for the full induction of several ISGs and is thus part of the innate immune response. This is supported by the observation that the transcriptional activity of ZNF395 depends on IKK signaling. Furthermore, the finding that the target genes of ZNF395 are involved in the innate immune response and cancer, implies that ZNF395 may support inflammation and cancer progression under hypoxia, which deserves further investigation.
